# Lead Exposure in Adult Males in Urban Transvaal Province, South Africa during the Apartheid Era

**DOI:** 10.1371/journal.pone.0058146

**Published:** 2013-03-07

**Authors:** Catherine A. Hess, Matthew J. Cooper, Martin J. Smith, Clive N. Trueman, Holger Schutkowski

**Affiliations:** 1 School of Applied Sciences, Bournemouth University, Talbot Campus, Poole, United Kingdom; 2 Ocean and Earth Sciences, National Oceanography Centre, University of Southampton, Southampton, United Kingdom; Stony Brook University, Graduate Program in Public Health, United States of America

## Abstract

Human exposure to lead is a substantial public health hazard worldwide and is particularly problematic in the Republic of South Africa given the country’s late cessation of leaded petrol. Lead exposure is associated with a number of serious health issues and diseases including developmental and cognitive deficiency, hypertension and heart disease. Understanding the distribution of lifetime lead burden within a given population is critical for reducing exposure rates. Femoral bone from 101 deceased adult males living in urban Transvaal Province (now Gauteng Province), South Africa between 1960 and 1998 were analyzed for lead concentration by Inductively Coupled Plasma Mass Spectrometry (ICP-MS). Of the 72 black and 29 white individuals sampled, chronic lead exposure was apparent in nearly all individuals. White males showed significantly higher median bone lead concentration (ME = 10.04 µg·g^−1^), than black males (ME = 3.80 µg·g^−1^) despite higher socioeconomic status. Bone lead concentration covaries significantly, though weakly, with individual age. There was no significant temporal trend in bone lead concentration. These results indicate that long-term low to moderate lead exposure is the historical norm among South African males. Unexpectedly, this research indicates that white males in the sample population were more highly exposed to lead.

## Introduction

Population-wide exposure to lead pollution is a problem that has, for the most part, been addressed in Europe and North America. However inorganic environmental pollution, specifically that of lead and other toxic heavy metals is a major public health concern in sub-Saharan Africa [1]–[5]. Lead in particular, is of growing concern because of its known toxicity at low levels. The problem of environmental lead pollution was largely overlooked in South Africa in the 20^th^ century, despite its substantial mining and industrial activities, and perhaps more surprisingly, despite the country’s persistent use of leaded petrol [1], [6]. Few studies of human lead exposure in Transvaal were published prior to the formation of the New South African Republic in 1994, which has left a prominent lack of baseline data with which to compare the growing body of public health research into the issue. Among the aims of this research is to address this gap in data through analysis of the skeletal remains of South African individuals who died before 1998. The Pretoria Bone collection, from which the study population derives, contains fully identified individuals and its use in this research comprises an unparalleled opportunity to study recent historical trends in human lead exposure. In addition, bone tissue is an endogenous repository for lead. Due to its low turnover rate compared to other human tissues – approximately 10 years for compact bone – bone lead concentration is an excellent indicator of chronic lead exposure [7]. Within Africa, the highly industrial Gauteng Province is among the more polluted regions. The region forms the backbone of the industrial and mining economy of South Africa and is home to the country’s most industrial city, Johannesburg and its capital, Pretoria. Urban pollution is a significant public health concern as is human exposure to lead [4], [5]. The results of this research may provide valuable background information to more recent studies involving human blood lead concentration in the region.

South Africa began monitoring lead exposure in children in the 1980s [8]–[11]. Studies conducted by von Schirnding et al. found that as many as 13 percent of children living in Cape Town had blood lead levels greater than or equal to 25 µg/dL – more than twice the threshold considered dangerous by the US Centers for Disease Control - and noted that proximity to traffic was a significant risk factor for elevated blood lead, as was lower socioeconomic status, overcrowding and homes in disrepair [10]–[12]. Deveaux et al. also conducted blood lead monitoring in young children in Cape Town and found that children whose blood lead was greater than or equal to 29 µg/dL were also living in homes with leaded paint [9].

Analysis of teeth from individuals buried in Cape Town before the introduction of leaded petrol show higher than expected lead concentrations which were also significantly higher than those measured in the mid-1980s and before the reduction in the lead concentration of petrol [13]. It was determined that the prevalent use of lead pipes in residential plumbing was responsible. To date only one study of bone lead has been conducted in South Africa. Todd et al. measured tibia lead in employees of a lead-acid battery factory [14]. They report a mean bone lead concentration of 53.4 µg·g^−1^.

Despite these early studies, as late as 2005 the country had no national lead monitoring program [15]. In addition, we could find no studies of lead exposure conducted in Transvaal during the apartheid era, leaving a gap in the understanding of the historical and demographic patterns associated with lead exposure. In addition, because of the cumulative nature of bone lead, this measure is widely considered to be a valuable indicator of chronic, as opposed to acute, lead exposure, and from an epidemiological standpoint, may be a more reliable indicator of demographic and long-term exposure patterns than blood lead [7], [16]–[18]. In light of these observations, this study aims to quantify lead exposure among urban South African males during apartheid by measuring bone lead concentration in an identified skeletal collection.

The authors wish to note that the racial terms “black” and “white” are used in this study to denote ethnic ancestry. This is wholly due to the fact that that the sampled population is classified in this way in associated cadaver records and because the population would have been segregated purely by racial classification during the time period being studied. These terms have social, demographic and political connotations the implications of which appear to have influenced patterns of lead exposure within the study population.

## Materials

Skeletal material was sampled from the Pretoria Identified Bone Collection at the University of Pretoria, South Africa and the Dart Student Bone Collection at Witwatersrand University, Johannesburg. The Pretoria Bone Collection is an identified reference collection held at the University of Pretoria, School of Medicine. The skeletal remains are those of individuals who died in the Pretoria area between 1943 and 2012 and whose bodies were either unclaimed or donated. In the former case, unclaimed bodies become the property of the University of Pretoria to be used for teaching and research, subject to the South Africa Human Tissues Act of 1983 [19]. The collection consists of individuals who range in age from neonates to 95 years of age. The predominant demographic within the collection is black males. This is largely to do with both overall demographic patterns within South Africa and to economic conditions during Apartheid, in which circulating migration brought black males to urban areas from Bantustans for work [19]–[21]. No information regarding the occupation of any of the individuals in either collection was available. The Raymond Dart Collection is housed at the University of Witwatersrand, School of Medicine and is similar in demographic composition to the Pretoria Collection. Skeletal remains in the Dart collection date to 1928 [22]. Only 12 of the femora included in this study are from the Dart collection. For both collections, ancestry was determined by the admitting hospital and based on the racial classification set forth in the 1950 Population Registration Act, which categorized individuals as black, white or colored based on physical appearance, parentage (an individual with one white and one black or colored parent could not be classified as white) and socio-cultural considerations. For the purpose of this paper, these classifications were not re-examined, as this research is primarily concerned with the way this racial division would have contributed to different lead exposure rates. In addition, because of the unique lack of fluidity between racial groups imposed by Apartheid, and because these groups largely defined socio-economic status at the time, the two factors are considered one and the same in this instance.

### Ethics Statement

This research was approved by the Bournemouth University Ethical Review Committee and the University of Pretoria, Department of Anatomy. In addition, the project met the requirements set by the UK Human Tissues Act (1994) and bone samples were imported into the UK and analyzed in accordance with the Act.

## Methods

### Analytical Methods

Cortical bone samples of approximately 0.250 g were removed from the right or left femora of 101 individuals who lived in Gauteng Province at the time of their death between 1961 and 1998. Bone samples were removed from femora with a 10 mm diamond-tipped core drill attached to a drill press. Cores were taken from the posterior-distal surface of the right or left femur, just above the intercondylar fossa and placed into sealed plastic bags until analysis. Due to the demographic composition of the collections which are biased heavily towards black males, the remains sampled were primarily black males. Analysis was conducted at the University of Southampton Geochemistry Class 100 Clean laboratory at the National Oceanography Centre Southampton. All reagents used were Fisher Trace Element grade and further sub-boiled in Teflon® stills to ensure ultra-purity. Water used was MilliQ® Millipore ultra-pure water (18.2 MΩ).

### Sample Preparation

Samples were weighed, washed three times with MilliQ® water to remove any surface contaminants and placed into acid-washed 13 mL polyethylene tubes. 1 mL of concentrated, sub-boiled HNO_3_ (69%) was added to each tube and left at room temperature for 72 hours. After initial digestion, 9 mL MilliQ® was added to each tube and samples were left to digest at room temperature (approx. 20°C) for a further 72 hours. To facilitate ICP-MS analysis, all samples were diluted to approximately 100 µg·g^−1^ calcium concentration with 3% sub-boiled HNO_3_.

### Sample Analysis

Samples were analyzed by ICP-MS (Thermo Scientific XSeries 2) calibrated with synthetic mixed element standards made from single element ICP-MS standards (Inorganic Ventures). All samples and standards contained 20 ng·g^−1^ Be and 5 ng·g^−1^ In and Re as internal standards. The elements were analyzed in one of two instrument modes depending on signal size and susceptibility to interferences. These were standard mode and CCT mode with 2 mL/min. of a mixed He/H_2_ gas added to reduce interferences. Ten reagent blanks of 3% HNO_3_ were analyzed and Pb concentration in all blanks was below the limit of detection. Detection limit for Pb is 0.0004 µg/L^−1^. Method validation was established by the inclusion of ten, 0.1 g samples of NIST SRM 1486 Bone Meal and Pb concentration is reported in Table 1. Mean Pb recovery rate in CRM was 90%. Sample duplicate precision was measured at 0.82 (*SD* = 0.32).

**Table 1:** Pb concentration and recovery rate for NIST 1486 Bone Meal.

Sample	Pb	% Recovery
	µg·g^−1^	
**NIST 1486**	1.33±.014	**–**
CRM 1	1.166	87.2
CRM2	1.034	78.0
CRM3	1.167	87.7
CRM4	1.217	92.0
CRM5	1.344	101.0
CRM7	1.229	92.4
CRM 8	1.248	75.1
CRM 10	1.259	95.0
**Avg.**		**88.5**

### Statistical Methods

Kolmogorov-Smirnov tests confirmed that bone Pb concentration was not normally distributed for either black males, *D*(74) = 0.255, p<0.001 or white males *D*(29) = 0.277, p = 0.001. Pb concentration data was log transformed and was found to be normally distributed with *D*(74) = 0.084, p>0.05 and *D*(29) = 0.133, p>0.05, in black and white males respectively. Independent t-tests, ANCOVA and multiple regression were performed on log-transformed data. All Pb concentrations reported are back-transformed values.

## Results

Median bone Pb by race and age group are presented in Table 2. There was a high degree of variability within the subject population as a whole. Of 72 black males, the median Pb concentration is 3.80 µg·g^−1^. For the 29 white males median Pb concentration is 10.04 µg·g^−1^. Results of Pb concentration for black males are presented in Table 3 and white males in Table 4. In some cases, samples from both right and left femora were taken from the same individual, in these cases Pb concentration in both femora within a single individual was averaged and is indicated by an asterisk.

**Table 2:** Median bone Pb (µg·g^−1^) in black and white urban South African males in relation to age.

Race		N	Pb	SD	IRQ	Min.	Max.
	**Black**	72	3.92	5.69	4.12	1.22	32.23
	**White**	29	10.04	13.61	9.58	1.55	64.09
	**Black**						
**Age**	20–29	9	2.22	2.7		1.7	9.25
	30–39	12	4.14	3.47		1.87	13.56
	40–49	18	3.3	2.48		1.22	11.56
	50–59	12	3.67	5.1		1.61	18.1
	60–69	11	4.53	1.72		1.9	6.73
	70–79	10	7.2	11.66		2.02	32.23
	80–89	1	12.95				
Median	49					18	80
	**White**						
	20–29	0					
	30–39	0					
	40–49	5	10.04	7.9		7.53	26.54
	50–59	7	10.85	7.02		6.38	27.6
	60–69	9	12.7	10.7		2.78	37
	70–79	3	3.41	12.56		1.55	24.18
	80–89	3	7.59	0.22		7.45	7.88
	90–99	2	49.07	21.25		34.04	64.1
Median	62					42	95

**Table 3:** Total bone lead concentration in µg·g^−1^ dry weight in femora of black males.

Specimen	Age	Death	Pb	Specimen	Age	Death	Pb
	(years)	(year)	µg·g^−1^		(years)	(year)	µg·g^−1^
2	30	1987	4.26	134	20	1964	1.80
3	38	1988	6.60	137	50	1965	14.71
4	36	1988	7.89	141	40	1961	2.19
5	67	1991	2.23	143	49	1966	4.49
6	40	1988	3.92	144	70	1969	7.63
7	30	1987	7.93	147	42	1979	2.72*
8	65	1985	4.76	148	48	1975	1.95
9	50	1988	3.45	150	61	1983	4.17
10	59	1987	18.05	151	65	1972	6.19
11	75	1988	32.23	152	50	1979	3.60
12	51	1991	6.14	156	55	1972	1.61
20	35	1988	2.51	158	50	1972	8.79
23	50	1983	2.95*	159	60	1982	4.53
29	30	1984	2.44	168	70	1979	6.74
48	30	1970	13.56	169	69	1983	1.85
51	58	1975	3.65	174	65	1979	6.73
61	56	1983	3.37	192	35	1967	1.87
63	48	1967	7.65	198	27	1964	6.00
64	40	1967	2.98	199	35	1966	2.48
79	45	1979	5.52	300	44	1979	3.53
83	40	1972	2.96	301	65	1965	2.72
86	44	1979	2.05	306	49	1976	3.05
88	24	1967	9.25	312	25	1966	2.13
89	80	1970	12.95	313	66	1967	6.02
90	70	1966	8.56	314	70	1966	15.66
92	56	1979	5.81	315	26	1972	3.08
95	47	1963	1.91	317	60	1983	4.95
99	40	1965	4.37	319	49	1967	11.56
101	48	1969	1.22	320	37	1966	4.36*
104	26	1966	1.69	321	43	1967	5.76
113	20	1979	2.18*	325	72	1979	2.99
115	70	1979	2.02	326	73	1980	32.13
121	40	1965	3.83	329	70	1983	3.93
123	59	1964	3.69	333	28	1965	6.46
125	70	1973	2.25	334	60	1982	2.38*
131	34	1970	4.03	335	18	1982	2.22

* Denotes averaged Pb concentration between right and left femora.

**Table 4:** Total bone lead concentration in µg·g^−1^ dry weight in femora of white males.

Specimen	Age	Death	Pb	Specimen	Age	Death	Pb
	(years)	(year)	µg·g^−1^		(years)	(year)	µg·g^−1^
17	67	1983	15.18	124	83	1993	7.59
39	62	1980	9.70	126	56	1982	8.30
59	84	1998	7.45	178	47	1977	7.53
60	71	1977	24.18	183	62	1983	12.70*
74	72	1998	3.40	185	68	1975	2.95
78	52	1983	6.38	190	66	1997	3.91
82	95	1982	64.09	191	74	1972	1.55
84	82	1997	7.88*	195	60	1973	2.78*
85	50	1976	13.26	295	44	1977	12.30
93	56	1979	9.34*	298	67	1976	37.00
94	42	1977	10.04	305	68	1964	18.44*
105	43	1964	26.54	322	69	1984	15.69
116	59	1982	10.85	324	57	1976	27.58
119	56	1982	12.15*	332	48	1973	7.82
120	91	1979	34.04				

* Denotes averaged Pb concentration between right and left femora.

**Figure 1:**
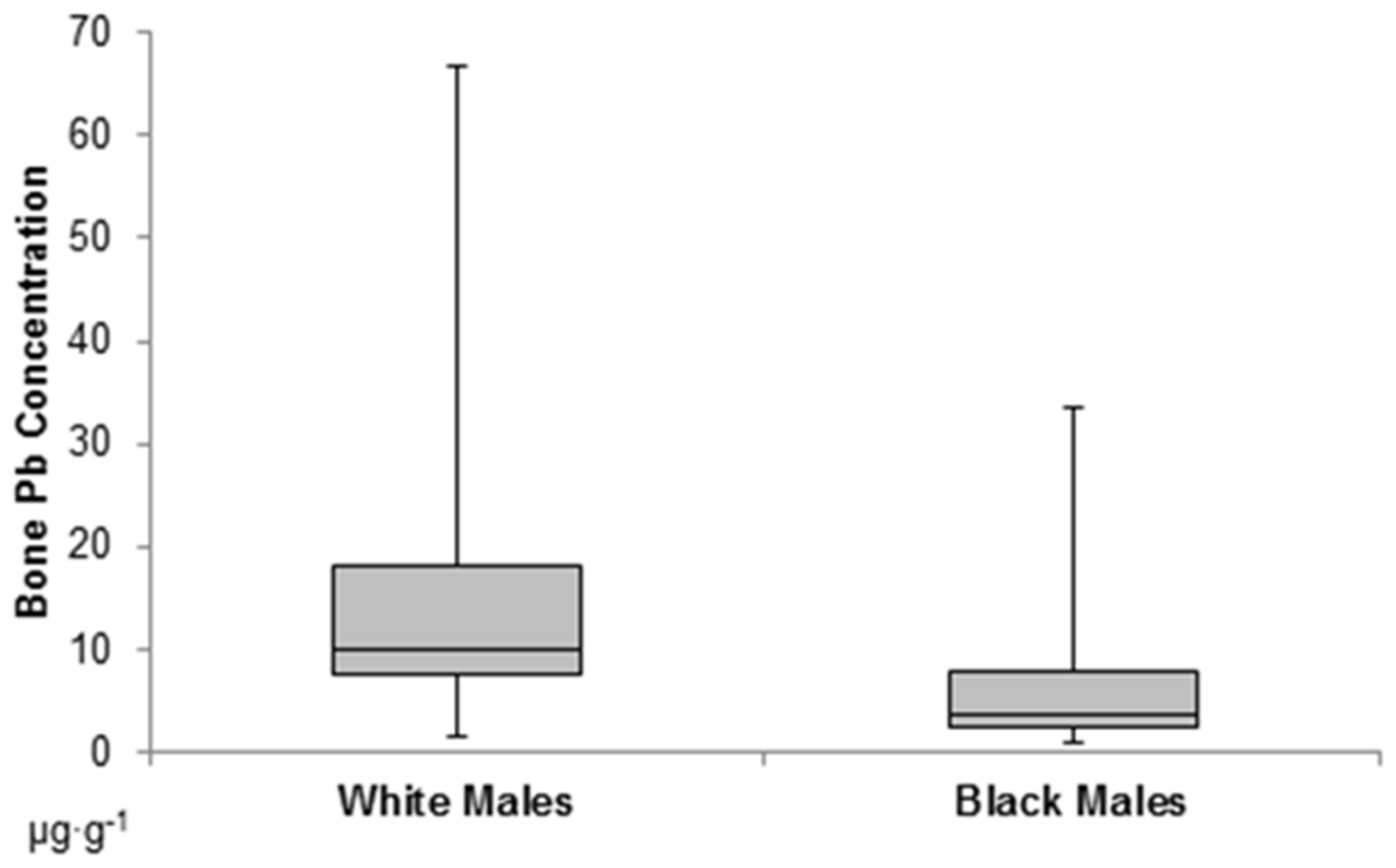
Boxplot of median bone Pb concentrations (in µg·g^−1^) in black and white males. White males show significantly higher bone Pb concentration than black males. Horizontal line = median, boxes = 2^nd^ and 3^rd^ quartiles, error bars = range.

White males show significantly higher bone Pb concentration than black males (Fig. 1). An independent t-test confirmed that the difference in means is significant, *t* (100) = 5.5, *p*<0.001. Among all samples, the highest individual concentrations occur in white males – samples 82 (64.09 µg·g^−1^) and 60 (24.8 µg·g^−1^). Among black males, the highest concentrations occur in samples 11 (32.23 µg·g^−1^) and 10 (18.05 µg·g^−1^).

There were significant difference in bone Pb concentrations between the 12 black males from the Dart Collection, and black males in the Pretoria Collection, *t* (71) = 2.23, *p*<0.05. Median bone Pb from males in the Dart collection is 6.14 µg·g^−1^ and 3.36 µg·g^−1^ in males from the Pretoria Collection. However this result must be accepted with caution, as there are only 12 samples from the Dart collection and there is considerable overlap in the admitting hospitals between the two collections, indicating that the individual remains in the two collections did not come from two distinct populations.

It is possible that some of the apparent differences in bone lead concentrations between black and white origin males could be attributed to age, as the white individuals are on average older than the black individuals (Table 2). Analysis of Covariance (ANCOVA) demonstrates that the covariate, age affects bone Pb concentration, *F* (1, 99) = 6.66, *p*<0.05, though the effect size is small, *r* = 0.06. After controlling for the effect of age, the effect of ancestry on bone Pb concentration remains significant, *F (*1, 99) = 19.20, *p*<0.001, though the effect size is relatively small, *r* = 0.145.

When multiple regression was used to test the relationship between age and ancestry, a significant linear trend between age, ancestry and bone Pb concentration is apparent. Both age and ancestry explain 28.4% of the variance in bone Pb concentration (R^2^ = .284, *F*(1, 102) = 19.82, p<.001). Both age and ancestry significantly predicts bone Pb (β = .235, p<.001 and β = .395, p>.001).

## Discussion

### Demographic Trends and Lead Exposure

The results of this study are particularly informative, in that they do not correspond to world-wide trends in human lead exposure. In general, and especially in developing countries, the poorest and most disadvantaged sectors within the population tend to encounter greater exposure to lead [23]–[25]. These groups also tend to yield the highest body concentrations of lead (bone or blood) [26], [27]. This is the case in present day South Africa. von Schirnding et al. [10] have reported blood lead levels among children residing in Cape Town, with children from lower income households having the highest levels. Mathee et al. [28] reported that high blood lead levels were associated with lower socioeconomic status in a study of children in Johannesburg. Other researchers have reported similar findings [2], [3].

The higher bone lead levels of white individuals reported in this study are interesting and may be the result of a variety of factors. The finding is counter to results of lead studies conducted in the United States. Research from the National Health and Nutrition Examination Surveys (NHANES) have consistently reported higher blood and bone lead concentrations in African American individuals regardless of age or sex [29], [30]. Data from the US Veterans’ Association Normative Aging Survey have also shown that white males tend to have lower bone and blood lead than African American males [31]. Similar results were found in the Baltimore Memory Study, in which authors reported significantly higher cortical bone lead in African American versus white males [32]. These patterns have persisted in the United States, even as overall lead exposure rates have fallen [33]. Most notably, Hu et al. [31] report a median bone lead concentration of 20 µg·g^−1^ in community exposed males living in Boston. This is higher than the median concentrations values found in South African males in this study. Hu et al. also report higher bone lead concentration in black males, and a significant increase in bone lead in males who did not complete high school versus those who completed graduate or professional school. The latter indicates a strong socioeconomic relationship with lead exposure.

With regards to our study, apartheid-mandated urban residential patterns, with white residents living closer to urban core and major roadways (with subsequent exposure to lead from petrol), may be the significant factors. Recent studies have reported greater atmospheric lead concentration in central business areas in Pretoria and Johannesburg, which during Apartheid were primarily white areas [34]–[37]. von Schirnding et al. found that atmospheric lead levels in the Cape Town city center were 2.5 times greater than in suburban areas [38]. The presence of lead paint in residential buildings and the possibility that homes built in the early 20^th^ century and before may be plumbed with lead pipes is another, though the latter appears to be rare [39]–[42]. Investigation into the source of lead in bone by analysis of lead isotopic ratios is currently underway, which may shed light on this phenomenon.

### Bone Lead and Age

This research confirms, though weakly, the previously reported association between age and bone lead concentration [43]–[45]. It has been estimated that 90% of the lead that is stored in the human body is stored in bone tissue [7], [46], [47]. This has the effect of sequestering lead from other tissues and organs where it may cause toxicity. However as individuals age and bone is resorbed, lead is released from bone tissue. The correlation between age and bone lead is well established and given the likelihood that bone acts as an endogenous source of lead within the body, releasing lead into the bloodstream as bone is resorbed and remodeled, it is clear that high lead levels in old age may have a significant impact on individual health [17].

### Bone Lead and Public Health

With regards to toxicity, it has been previously reported that bone lead levels as low as 5 µg·g^−1^ have been associated with clinical symptoms of toxicity such as hypertension [48]–[52]. In this study, 38% of black individuals and 86% of white individuals had bone lead levels above this threshold. Overall, however, the bone lead concentration in males in this study population is relatively moderate. Baranowska et al. [53] reported bone lead levels between 100 and 200 µg·g^−1^ in an industrial district in Poland. Nevertheless, in the past decade it has become increasingly clear that chronic low-level exposure to lead is a substantial threat to individual and public health [54].

Reported health effects of chronic lead exposure include renal disease, diminished IQ and developmental delay (in children), and impaired cognitive function in adults [55]–[64]. Most recently, the drop in violent crime rates in urban areas in the United States has been attributed to the fall in lead pollution following the banning of lead in petrol [56], [65], [66]. Many of these pathologies are evident even at the subclinical level and at relatively low levels of exposure. Norman et al. [51] report that in South Africa in 2000, nearly 1,500 deaths could be attributed directly to lead exposure. Other studies have found that low-level lead exposure in men leads to diminished cognitive function on the order of five years accelerated mental aging [67]. From the results of this study, it is likely the negative effects of lead on public health have been acting on the population for some time. In addition, the data above suggest that persistent lower-level exposure to lead may be the norm in South Africa (even after the cessation of the use of lead in petrol). This low but chronic level of exposure may be particularly pernicious, as subclinical or sub-acute symptoms are often overlooked in marginalised populations due, in part, to differential access to medical care and lifestyle [68]–[70]. Potentially then, despite lower lead exposure overall, black males may be more susceptible to unfavourable health effects.

It is critical to acknowledge that, though black individuals may show lower bone lead concentrations, the burden of disease resulting from lead may be higher in this demographic group. Numerous studies have demonstrated that individuals who may be physically or nutritionally stressed are also likely to suffer from the effects of lead toxicity at lower exposure levels than healthier individuals [71]–[73]. Within these populations lead exposure may also be associated with other illnesses such as asthma and iron deficiency anemia, both of which are prevalent in low income households in South Africa [74]–[78].

In summary, bone lead analysis of apartheid-era skeletal remains has yielded unexpected results. White males show significantly higher bone lead concentration than black males. This difference could be attributed to use of exposure to leaded petrol and exacerbated by residential patterns in urban areas in which white individuals resided closer to the congested urban core.

## References

[1] Diab RD (1999) A note on changes in atmospheric lead content in seven cities in South Africa. South African Journal of Science 95: 117–121.

[2] Harper CC, Mathee A, von Schirnding Y, De Rosa CT, Falk H (2003) The health impact of environmental pollutants: a special focus on lead exposure in South Africa. International Journal of Hygiene and Environmental Health 206: 315–322. 12971686 10.1078/1438-4639-00227

[3] Nriagu JO (1992) Toxic metal pollution in Africa. The Science of The Total Environment 121: 1–37. 1439723 10.1016/0048-9697(92)90304-b

[4] Nriagu JO, Blankson ML, Ocran K (1996) Childhood lead poisoning in Africa: a growing public health problem. Science of The Total Environment 181: 93–100. 8820380 10.1016/0048-9697(95)04954-1

[5] Nweke OC, Sanders WH (2009) Modern Environmental Health Hazards: A Public Health Issue of Increasing Significance in Africa. Environmental Health Perspectives 117: 863–870. 19590675 10.1289/ehp.0800126 PMC2702398

[6] Mathee A, Rollin H, von Schirnding Y, Levin J, Naik I (2006) Reductions in blood lead levels among school children following the introduction of unleaded petrol in South Africa. Environmental Research 100: 319–322. 16213479 10.1016/j.envres.2005.08.001

[7] Rabinowitz MB (1991) Toxicokinetics of bone lead. Environmental Health Perspectives 91: 33–37. 2040248 10.1289/ehp.919133 PMC1519353

[8] White NW, Dempster WS, Pocock F, Kibel MA (1982) Lead absorption in Cape children - a preliminary report. South African Medical Journal 62: 799–802. 7147106

[9] Deveaux P, Kibel MA, Dempster WS, Pocock F, Formenti K (1986) Blood lead levels in pre-school children in Cape Town. SAMJ-South African Medical Journal 69: 421–424. 3961631

[10] von Schirnding Y (1991) Blood lead levels in South African inner-city children. Environmental Health Perspectives 94: 125–130. 1720096 10.1289/ehp.94-1567937 PMC1567937

[11] von Schirnding Y, Fuggle RF, Bradshaw D (1991) Factors associated with elevated blood lead levels in inner-city Cape Town children. South African Medical Journal 79: 454–456. 2020886

[12] Centers for Disease Control (2005) Preventing Lead Poisoning in Young Children. Atlanta: CDC.

[13] Grobler SR, Theunissen FS, Maresky LS (1996) Evidence of undue lead exposure in Cape Town before the advent of leaded petrol. South African Journal of Medicine 86: 169–171. 8619146

[14] Todd AC, Ehrlich RI, Selby P, Jordaan E (2000) Repeatability of Tibia Lead Measurement by X-Ray Fluorescence in a Battery-Making Workforce. Environmental Research 84: 282–289. 11097802 10.1006/enrs.1999.4090

[15] Mathee A, von Schirnding Y, Montgomery M, Rollin H (2004) Lead poisoning in South African children: the hazard is at home. Reviews on Environmental Health 19: 347–361. 15742678

[16] Bergdahl IA, Skerfving S (2008) Biomonitoring of lead exposure-alternatives to blood. Journal of Toxicology and Environmental Health-Part A-Current Issues 71: 1235–1243. 10.1080/15287390802209525 18654894

[17] Hu H, Rabinowitz M, Smith D (1998) Bone lead as a biological marker in epidemiologic studies of chronic toxicity: Conceptual paradigms. Environmental Health Perspectives 106: 1–8. 9417769 10.1289/ehp.981061 PMC1532948

[18] Hu H (1998) Bone lead as a new biologic marker of lead dose: Recent findings and implications for public health. Environmental Health Perspectives 106: 961–967. 9703479 10.1289/ehp.98106s4961 PMC1533327

[19] L’Abbe E, Loots M, Meiring JH (2005) The Pretoria Bone Collection: A modern South African Skeletal Sample. Homo 56: 197–205. 16130841 10.1016/j.jchb.2004.10.004

[20] Byerlee D (1974) Rural - urban immingration in Africa - Theory, policy and research implications. International Migration Review 8: 543–566.

[21] Smit R (2001) The impact of labor migration on African families in South Africa: Yesterday and today. Journal of Comparative Family Studies 32: 533.

[22] Dayal MR, Kegley ADT, Štrkalj G, Bidmos MA, Kuykendall KL (2009) The history and composition of the Raymond A. Dart Collection of Human Skeletons at the University of the Witwatersrand, Johannesburg, South Africa. American Journal of Physical Anthropology 140: 324–335. 19382178 10.1002/ajpa.21072

[23] Brown RW, Longoria T (2010) Multiple Risk Factors for Lead Poisoning in Hispanic Sub-Populations: A Review. Journal of Immigrant and Minority Health 12: 715–725. 19330449 10.1007/s10903-009-9245-8

[24] Grosse SD, Matte TD, Schwartz J, Jackson RJ (2002) Economic gains resulting from the reduction in children’s exposure to lead in the United States. Environmental Health Perspectives 110: 563–569. 12055046 10.1289/ehp.02110563 PMC1240871

[25] Pirkle JL, Brody DJ, Gunter EW, Kramer RA, Paschal DC, et al (1994) The Decline in Blood Lead Levels in the United States: The National Health and Nutrition Examination Surveys (NHANES). JAMA: The Journal of the American Medical Association 272: 284–291. 8028141

[26] Ezzati M, Lopez AD, Rodgers A, Vander Hoorn S, Murray CJL, et al (2002) Selected major risk factors and global and regional burden of disease. Lancet 360: 1347–1360. 12423980 10.1016/S0140-6736(02)11403-6

[27] Thomas KJA (2007) Child mortality and socioeconomic status: An examination of differentials by migration status in South Africa. International Migration Review 41: 40–74.

[28] Mathee A, von Schirnding YER, Levin J, Ismail A, Huntley R, et al (2002) A survey of blood lead levels among young Johannesburg school children. Environmental Research 90: 181–184. 12477462 10.1016/s0013-9351(02)00010-5

[29] Harlan WR (1988) The relationship of blood lead levels to blood pressure in the US population. Environmental health perspectives 78: 9. 3203651 10.1289/ehp.88789 PMC1474598

[30] Lanphear BP, Weitzman M, Eberly S (1996) Racial differences in Urban children’s environmental exposures to lead. American journal of public health 86: 1460–1463. 8876521 10.2105/ajph.86.10.1460 PMC1380663

[31] Hu H, Payton M, Kornc S, Aro A, Sparrow D, et al (1996) Determinants of Bone and Blood Lead Levels among Community-exposed Middle-aged to Elderly Men: The Normative Aging Study. American Journal of Epidemiology 144: 749–759. 8857824 10.1093/oxfordjournals.aje.a008999

[32] Martin D, Glass TA, Bandeen-Roche K, Todd AC, Shi W, et al (2006) Association of Blood Lead and Tibia Lead with Blood Pressure and Hypertension in a Community Sample of Older Adults. American Journal of Epidemiology 163: 467–478. 16421242 10.1093/aje/kwj060

[33] Muntner P, Madkbrfab V (2005) Continued decline in blood lead levels among adults in the United States: The National Health and Nutrition Examination Surveys. Archives of Internal Medicine 165: 2155–2161. 16217007 10.1001/archinte.165.18.2155

[34] Monna F, Poujol M, Losno R, Dominik J, Annegarn H, et al (2006) Origin of atmospheric lead in Johannesburg, South Africa. Atmospheric Environment 40: 6554–6566.

[35] Olowoyo JO, van Heerden E, Fischer JL, Baker C (2010) Trace metals in soil and leaves of Jacaranda mimosifolia in Tshwane area, South Africa. Atmospheric Environment 44: 1826–1830.

[36] Olowoyo JO, van Heerden E, Fischer JL (2011) Trace element concentrations from lichen transplants in Pretoria, South Africa. Environmental Science and Pollution Research 18: 663–668. 21080091 10.1007/s11356-010-0410-3

[37] Lemon A (1991) Homes apart : South Africa’s segregated cities. London: Chapman.

[38] Von Schirnding YER, Fuggle RF (1996) A study of the distribution of urban environmental lead levels in Cape Town, South Africa. Science of The Total Environment 188: 1–8. 8848712 10.1016/0048-9697(96)05122-4

[39] Von Schirnding YER, Fuggle RF, Bradshaw D (1991) Factors associated with elevated blood lead levels in inner-city Cape Town children. SAMJ-South African Medical Journal 79: 454–456. 2020886

[40] Mathee A, Rollin H, Levin J, Naik I (2006) Lead in Paint: Three Decades Later and Still a Hazard for African Children? Environ Health Perspect 115: 321–322. 17431477 10.1289/ehp.9575 PMC1849931

[41] Mathee A, Singh E, Mogotsi M, Timothy G, Maduka B, et al (2009) Lead-based paint on playground equipment in public children’s parks in Johannesburg, Tshwane and Ekurhuleni. SAMJ- South African Medical Journal 99: 819–821. 20218484

[42] Montgomery M, Mathee A (2005) A preliminary study of residential paint lead concentrations in Johannesburg. Environmental Research 98: 279–283. 15910783 10.1016/j.envres.2004.10.006

[43] Morrow L, Needleman HL, McFarland C, Aethenv K, Tobin M (2007) Past occupational exposure to lead: Association between current blood lead and bone lead. Archives of Environmental & Occupational Health 62: 183–186. 18458021 10.3200/AEOH.62.4.183-186

[44] Barry PSI, Mossman DB (1970) Lead concentrations in human tissues. British Journal of Industrial Medicine 27: 339–351. 5488693 10.1136/oem.27.4.339 PMC1069425

[45] Barry PS (1975) A comparison of concentrations of lead in human tissues. British Journal of Industrial Medicine 32: 119–139. 1131339 10.1136/oem.32.2.119 PMC1008038

[46] Wittmers LE, Wallgren J, Alich A, Aufderheide AC, Rapp G (1988) Lead in Bone IV. Distribution of lead in the human skeleton. Archives of Environmental Health 43: 381–391. 3196073 10.1080/00039896.1988.9935855

[47] Rabinowitz MB, Wetherill GW, Kopple JD (1976) Kinetic analysis of lead metabolism in healthy humans. The Journal of Clinical Investigation 58: 260–270. 783195 10.1172/JCI108467 PMC333178

[48] Cheng YW, Schwartz J, Sparrow D, Aro A, Weiss ST, et al (2001) Bone lead and blood lead levels in relation to baseline blood pressure and the prospective development of hypertension - The Normative Aging Study. American Journal of Epidemiology 153: 164–171. 11159162 10.1093/aje/153.2.164

[49] Glenn BS, Bandeen-Roche K, Lee BK, Weaver VM, Todd AC, et al (2006) Changes in systolic blood pressure associated with lead in blood and bone. Epidemiology 17: 538–544. 16906055 10.1097/01.ede.0000231284.19078.4b

[50] Navas-Acien A, Schwartz BS, Rothenberg SJ, Hu H, Silbergeld EK, et al (2008) Bone lead levels and blood pressure endpoints. Epidemiology 19: 496–504. 18414090 10.1097/EDE.0b013e31816a2400

[51] Norman R, Mathee A, Barnes B, van der Merwe L, Bradshaw D, et al (2007) Estimating the burden of disease attributable to lead exposure in South Africa in 2000. SAMJ -South African Medical Journal 97: 773–780. 17952236

[52] Wedeen RP (1988) Bone lead, hypertension, and lead nephropathy. Environmental Health Perspectives 78: 57–60. 3203647 10.1289/ehp.887857 PMC1474623

[53] Baranowska I, Czernicki K, Aleksandrowicz R (1995) The analysis of lead, cadmium, zinc, copper and nickel content in human bones from the Upper Silesian Industrial District. Science of the Total Environment 159: 155–162. 7878447 10.1016/0048-9697(95)04218-p

[54] Needleman H (2004) Lead poisoning. Annual Review of Medicine 55: 209–222. 10.1146/annurev.med.55.091902.103653 14746518

[55] Baghurst PA, McMichael AJ, Wigg NR, Vimpani GV, Robertson EF, et al (1992) Environmental Exposure to Lead and Children’s Intelligence at the Age of Seven Years. New England Journal of Medicine 327: 1279–1284. 1383818 10.1056/NEJM199210293271805

[56] Carpenter DO, Nevin R (2010) Environmental causes of violence. Physiology & Behavior 99: 260–268. 19758571 10.1016/j.physbeh.2009.09.001

[57] Koller Ka (2004) Recent developments in low-level lead exposure and intellectual impairment in children. Environmental Health Perspectives 112: 987–994. 15198918 10.1289/ehp.6941 PMC1247191

[58] Needleman HL, Riess JA, Tobin MJ, Biesecker GE, Greenhouse JB (1996) Bone lead levels and delinquent behavior. JAMA-Journal of the American Medical Association 275: 363–369. 8569015

[59] Needleman HL, McFarland C, Ness RB, Fienberg SE, Tobin MJ (2002) Bone lead levels in adjudicated delinquents - A case control study. Neurotoxicology and Teratology 24: 711–717. 12460653 10.1016/s0892-0362(02)00269-6

[60] Nevin R (2000) How lead exposure relates to temporal changes in IQ, violent crime, and unwed pregnancy. Environmental Research 83: 1–22. 10845777 10.1006/enrs.1999.4045

[61] Nevin R (2009) Trends in preschool lead exposure, mental retardation, and scholastic achievement: Association or causation? Environmental Research 109: 301–310. 19167707 10.1016/j.envres.2008.12.003

[62] Bellinger DC, Stiles KM, Needleman HL (1992) Low level lead exposure, intelligence and academic achievement - a long term follow up study. Pediatrics 90: 855–861. 1437425

[63] Bandeen-Roche K, Glass TA, Bolla KI, Todd AC, Schwartz BS (2009) Cumulative Lead Dose and Cognitive Function in Older Adults. Epidemiology 20: 831–839. 19752734 10.1097/EDE.0b013e3181b5f100 PMC3523304

[64] van Wijngaarden E, Campbell JR, Cory-Slechta DA (2009) Bone lead levels are associated with measures of memory impairment in older adults. Neurotoxicology 30: 572–580. 19477197 10.1016/j.neuro.2009.05.007 PMC2719051

[65] Wright JP, Boisvert D, Vaske J (2009) Blood Lead Levels in Early Childhood Predict Adulthood Psychopathy. Youth Violence and Juvenile Justice 7: 208–222.

[66] Mielke HW, Zahran S (2012) The urban rise and fall of air lead (Pb) and the latent surge and retreat of societal violence. Environment International 43: 48–55. 22484219 10.1016/j.envint.2012.03.005

[67] Weisskopf MG, Wright RO, Schwartz J, Spiro A, Sparrow D, et al (2004) Cumulative lead exposure and prospective change in cognition among elderly men. American Journal of Epidemiology 160: 1184–1193. 15583371 10.1093/aje/kwh333

[68] Kon ZR, Lackan N (2008) Ethnic Disparities in Access to Care in Post-Apartheid South Africa. American Journal of Public Health 98: 2272–2277. 18923120 10.2105/AJPH.2007.127829 PMC2636545

[69] Peltzer K (2001) Psychosocial correlates of healthy lifestyles in black and white South Africans. Social Behavior and Personality 29: 249–255.

[70] Peltzer K (2002) Health behaviour among Black and White South Africans. Journal of the Royal Society for the Promotion of Health 122: 187–193. 12391834 10.1177/146642400212200316

[71] Herman DS, Geraldine M, Venkatesh T (2009) Influence of minerals on lead-induced alterations in liver function in rats exposed to long-term lead exposure. Journal of Hazardous Materials 166: 1410–1414. 19167163 10.1016/j.jhazmat.2008.12.070

[72] Mahaffey KR (1983) Biotoxicity of lead - Influence of various factors. Federation Proceedings 42: 1730–1734. 6832395

[73] Goyer RA (1995) Nutrition and metal toxicity. American Journal of Clinical Nutrition 61: S646–S650. 10.1093/ajcn/61.3.646S 7879732

[74] Motosue AM, Petronella S, Sullivan J, Castillo S, Garcia T, et al (2009) Lead Exposure Risk is Associated with Asthma in a Low-income Urban Hispanic Population: Results of the Communities Organized against Asthma and Lead (COAL) Project. Journal of Allergy and Clinical Immunology 123: S20.

[75] van Gemert F, van der Molen T, Jones R, Chavannes N (2011) The impact of asthma and COPD in sub-Saharan Africa. Primary Care Respiratory Journal 20: 240–248. 10.4104/pcrj.2011.00027 PMC6549843 21509418

[76] Heckman J, Samie A, Bessong P, Ntsieni M, Hamandi H, et al (2010) Anaemia among clinically well under-fives attending a community health centre in Venda, Limpopo Province. SAMJ -South African Medical Journal 100: 445–448. 20822592 10.7196/samj.3579 PMC2936714

[77] Faber M, Wenhold F (2007) Nutrition in contemporary South Africa. Water Sa 33: 393–400.

[78] Nojilana B, Norman R, Dhansay MA, Labadarios D, van Stuijvenberg ME, et al (2007) Estimating the burden of disease attributable to iron deficiency anaemia in South Africa in 2000. SAMJ-South African Medical Journal 97: 741–746. 17952232

